# Helicopter inter-hospital transfer for patients undergoing extracorporeal membrane oxygenation: a retrospective 12-year analysis of a service system

**DOI:** 10.1186/s13049-022-01018-0

**Published:** 2022-05-07

**Authors:** Alexander Fuchs, Renate Schmucki, Lorenz Meuli, Pedro David Wendel-Garcia, Roland Albrecht, Robert Greif, Urs Pietsch

**Affiliations:** 1 Department of Anaesthesiology and Pain Medicine, Inselspital, Bern University Hospital, University of Bern, Freiburgstrasse, 3010 Bern, Switzerland; 2 Department of Anaesthesiology and Intensive Care Medicine, Cantonal Hospital St. Gallen, St. Gallen, Switzerland; 3 Department of Vascular Surgery, University Hospital Zurich, Zurich, Switzerland; 4 Institute of Intensive Care Medicine, University Hospital Zurich, Zurich, Switzerland; 5 Swiss Air-Rescue (Rega), Zurich, Switzerland; 6 School of Medicine, Sigmund Freud University Vienna, Vienna, Austria; 7 European Resuscitation Council (ERC) Research NET, Niel, Belgium; 8 Department of Emergency Medicine, Inselspital, Bern University Hospital, University of Bern, Bern, Switzerland

**Keywords:** Extracorporeal membrane oxygenation, Extracorporeal life support, Inter-hospital transfer, Air-medical transport, Helicopter emergency medical system, Adverse events

## Abstract

**Background:**

Patients undergoing extracorporeal membrane oxygenation (ECMO) are critically ill and show high mortality. Inter-hospital transfer of these patients has to be safe, with high survival rates during transport without potentially serious and life-threatening adverse events. The Swiss Air-Rescue provides 24-h/7-days per week inter-hospital helicopter transfers that include on-site ECMO cannulation if needed. This retrospective observational study describes adverse events of patients on ECMO transported by helicopter, and their associated survival.

**Methods:**

All patients on ECMO with inter-hospital transfer by helicopter from start of service in February 2009 until May 2021 were included. Patients not transported by helicopter or with missing medical records were excluded. Patient demographics (age, sex) and medical history (type of and reason for ECMO), mission details (flight distance, times, primary or secondary transport), adverse events during the inter-hospital transfer, and survival of transferred patients were recorded. The primary endpoint was patient survival during transfer. Secondary endpoints were adverse events during transfer and 28-day survival.

**Results:**

We screened 214 ECMO-related missions and included 191 in this analysis. Median age was 54.6 [IQR 46.1–62.0] years, 70.7% were male, and most patients had veno-arterial ECMO (56.5%). The main reasons for ECMO were pulmonary (46.1%) or cardiac (44.0%) failure. Most were daytime (69.8%) and primary missions (n = 100), median total mission time was 182.0 [143.0–254.0] min, and median transfer distance was 52.7 [33.2–71.1] km. All patients survived the transfer. Forty-four adverse events were recorded during 37 missions (19.4%), where 31 (70.5%) were medical and none resulted in patient harm. Adverse events occurred more frequently during night-time missions (59.9%, *p* = 0.047). Data for 28-day survival were available for 157 patients, of which 86 (54.8%) were alive.

**Conclusion:**

All patients under ECMO survived the helicopter transport. Adverse events were observed for about 20% of the flight missions, with a tendency during the night-time flights, none harmed the patients. Inter-hospital transfer for patients undergoing ECMO provided by 24-h/7-d per week helicopter emergency medical service teams can be considered as feasible and safe. The majority of the patients (54.8%) were still alive after 28 days.

**Supplementary Information:**

The online version contains supplementary material available at 10.1186/s13049-022-01018-0.

## Background

Extracorporeal membrane oxygenation (ECMO) is a life-saving procedure during pulmonary and cardiac failure. The mortality of patients undergoing ECMO depends on the underlying medical cause, co-morbidities and age. Neonatal mortality under veno-venous ECMO is about 12% [1], while adult mortality (mostly on veno-arterial ECMO) has been reported from 54 to 64% [2, 3]. Systematic reviews have reported adverse events during cannulation and treatment in up to 21% of patients for neonates, and up to 52% for adults [1, 2]. In neonates, most complications are pneumothorax, hypertension and cannula dysfunction; in adults these are renal failure, pneumonia and bleeding [1, 2]. According to the 2021 Extracorporeal Life Support Organization (ELSO) registry report, in 2020 there were 521 centres with a total of 18,260 ECMO runs [4]. In this registry, the pooled adult and paediatric patient survival rates to discharge or transfer from 1990 to 2020 was reported to be 54%. An emerging indication for ECMO is for patients with cardiac arrest, which is called extracorporeal cardiopulmonary resuscitation (ECPR). The European Resuscitation Council first introduced ECPR in their 2015 advanced life support guidelines, and it remains in their 2021 guidelines [5]. A recent systematic review of the effectiveness of ECPR in out-of-hospital cardiac arrest that included data on over 3000 patients indicated that ECPR increases the survival in this cohort [6].

The special medical equipment and high expertise of ECMO require specialised ‘high-quality’ tertiary critical care centres [7]. A high case load of ECMO contributed to improved patient survival in a paediatric population [8]. Some centres offer special ECMO retrieval teams [9–11]. These teams implement on-site ECMO in the referral hospital (“primary mission”) and transport patients on ECMO to the tertiary care centre for further treatment. “Secondary missions” transfer patients who are already on ECMO to another hospital. The medical indications for an inter-hospital transfer must be carefully considered, as such transfers on ECMO can expose these vulnerable patients to additional risk of adverse events, or even death [10, 11]. Data on adverse events during ECMO transport are rare, with reports of 27% to 32% of patients suffering adverse events during transport [10–12].

Air-medical inter-hospital transfer is considered safe [13], but fatal outcomes have been reported [11, 12]. As this transport is highly complex and time consuming [10], the ELSO provides specific guidelines for ECMO transport [14].

The Swiss Air-Rescue provides a 24-h/7-days per week helicopter emergency medical system (HEMS) for national and international pre-hospital emergencies from different bases, and for inter-hospital transfers that include ECMO transfers. The evidence on the quality and safety of such a 24-h/7-days per week HEMS ECMO transfer services are rare. Therefore, this retrospective observational trial aimed to report on adverse events and survival rates of patients under ECMO during and following helicopter inter-hospital transfer.

## Methods

### Study and patients

The study protocol was approved by the Ethics Committee of Eastern Switzerland (EKOS 21/064, St. Gallen, Switzerland), and due to the retrospective study design and anonymised character of the data, the need for informed consent was waived by the Ethics Committee. All of the patient data were extracted from the Swiss Air-Rescue information system, and anonymised into an electronic research database. The study was performed according to the Declaration of Helsinki and the Swiss Act on Human Research. The reporting follows the Strengthening the Reporting of Observational Studies in Epidemiology (STROBE) guidelines [15].

The Swiss tertiary hospitals in Bern, Geneva, Lausanne, St. Gallen and Zurich offer an ECMO retrieval service, with the availability of cardiovascular surgeons or intensivists and perfusionists. The helicopter crew consists of a pilot, a flight paramedic and a board-certified specialist in anaesthesia or intensive care medicine who has additional pre-hospital emergency medicine training. There is no change in staffing composition and seniority regarding the time of day. The helicopter fleet for ECMO transfers comprises Airbus H145, all are equipped with avionics permitting night operations with and without night vision goggles under visual flight rules, but also under instrument flight rules. The standard ECMO device used was the Cardiohelp System (Maquet, Rastatt, Germany), and the helicopter was equipped with a custom-made fixation plate (Getinge, Rastatt, Germany) to avoid dislocation of the device during the flight. According to the ELSO guidelines [14], we defined primary missions when ECMO was implanted in the referral hospital by the retrieval ECMO team. In such cases, the HEMS crew was accompanied by a cardiovascular surgeon and a perfusionist. Secondary missions were defined as transport of patients with ECMO installed, and the HEMS crew was accompanied only by an additional perfusionist.

All patients undergoing ECMO who were transferred by helicopter from 1 February 2009 (start of service) until 30 April 2021 were included. Missions that only transported the ECMO team without a patient, missions without medical records available and non-helicopter transfers were excluded.

### Measurements

The patient data were extracted from the mission transport protocols, which included demographic data (age, sex), medical history and treatment (diagnosis, reason for ECMO, type of ECMO, medication, and additional devices), mission details (flight distance, flight-time, total mission time). Flights between 06:00 h and 20:00 h were classified as daytime missions, flights between 20:01 h and 05:59 h were classified as night-time missions. Mission times were defined as:

- response time, from alert of helicopter crew to landing at referral hospital
- Flight time, helicopter start at referral hospital with patient to landing at destination hospital
- Handover time, helicopter landing at referral hospital to take-off with patient to destination hospital
- Total mission time, alert of helicopter crew to landing at destination hospital with patient

The main medical diagnoses were coded according to the 2019 World Health Organisation International Classification of Diseases (ICD-10) [16]. The reasons for ECMO were categorised into four main categories by the authors based on the main medical diagnoses of: (a) pulmonary, (b) cardiac, (c) combined, or (d) other. Two of the authors (AF, RS) screened the transport protocols independently for documented adverse events during the inter-hospital transfer, and investigated potential non-documented adverse events (e.g., sudden change in heart rate, blood pressure, intravenous fluids, peripheral oxygen saturation, or ECMO flow). The adverse events were further classified into medical (e.g., medical condition or treatment associated) and non-medical (e.g., organization or weather-related). Patient survival during the transport was documented for all patients in the transport protocol. Follow-up data were available according to the patient medical records for 28-day survival or Intensive Care Unit discharge, and survival to hospital discharge was also recorded. The primary endpoint of the study was the survival of the patient during the transfer, and the secondary endpoints were adverse events during the transfer and 28-day survival.

### Statistics

Statistical analysis was performed using R environment version 4.0.2 [17]. Due to the observational character of the study, no formal sample size calculation was performed. Continuous variables were summarised by *median* and the first and third quartiles (*Q1*, *Q3*, respectively), if skewed. Data was inspected for normality using histograms and formally tested using the Shapiro–Wilk tests. Normally distributed data was summarized by mean and standard deviation. Categorical variables were summarised by counts and percentage for each level of the variable, and compared using Pearson’s chi-squared tests. Continuous variables were compared using student’s t-tests if they were normally distributed, or using Kruskal–Wallis rank tests if they were skewed. P-values are two-sided with an α-level of 5%.

To analyse the factors associated with complications during transportation and the in-hospital mortality, multivariable logistic regression models were built that included the variables daytime (binary; from 06:00 h to 20:00 h), sex, age (years), total mission time (minutes), type of ECMO (veno-venous vs. veno-arterial or veno-venoarterial). Veno-venoarterial was added to the veno-arterial group due to the low number of patients (n = 2), and diagnosis group (pulmonary vs. cardiac vs. combined vs. other). Multicollinearity was checked by correlation coefficients. Multicollinearity was accepted up to a correlation of coefficients of 0.7. Multicollinear variables were eliminated based on clinical decisions. Interactions between covariates were tested and removed if they did not improve model fit.

Global positioning system coordinates were used for geographic plotting and analysis of the direct distances between the hospitals (as the approximation for flight distance). The topographic relief was visualised using swisstopo data from 2016 [18].

## Results

**Fig. 1:**
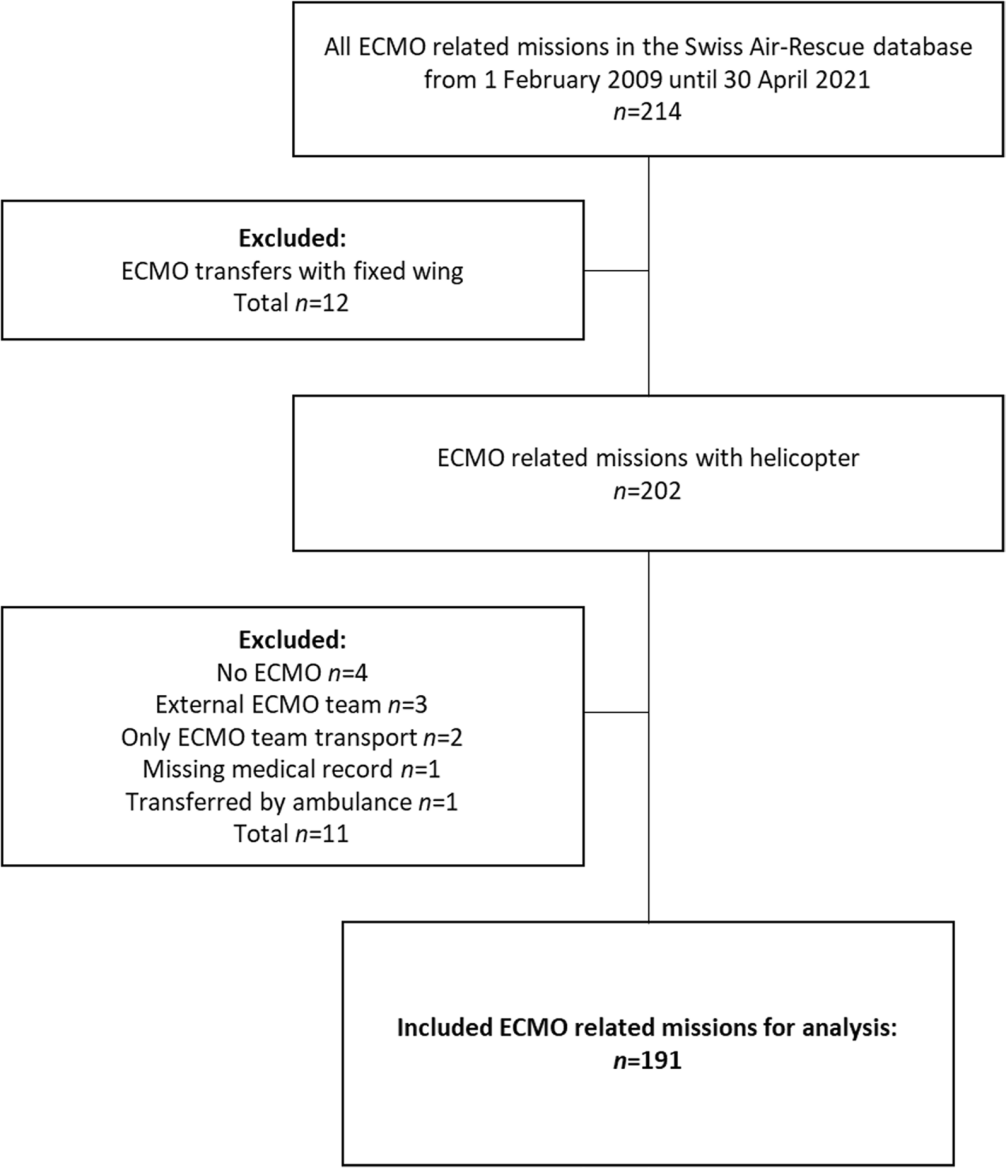
Study flow chart

We screened 214 ECMO-related missions during these 12 years of the service, and 191 were included in the final analysis, as shown in Fig. 1. More than two-thirds were daytime transfers (134, 70.1%). Detailed patient demographics and medical history details are given in Table 1. Six missions were for patients under 18 years of age. The main reasons for ECMO were pulmonary (88, 45.8%) or cardiac (84, 44.0%) failure. Over half of the patients were treated with veno-arterial ECMO (108, 56.5%). Thirty-five patients (18.3%) had ECPR. Details on the medical diagnoses are reported in the Additional file 1. The majority of patients were transported intubated (180, 93.8%) and sedated (173, 90.1%), and had at least one vasopressor (162, 84.4%).

**Table 1:** Demographics and medical history data for transported patients undergoing extracorporeal membrane oxygenation (ECMO)

Demographics/medical history	Daytime	Night-time	Total	*p*
Numbers [n (%)]	**134 (70.2)**	**57 (29.8)**	**191 (100.0)**	
Male [n (%)]	101 (75.4)	34 (59.6)	135 (70.7)	**0.029**
Age (year; median [Q1–Q3])	55.1 [46.5–62.3]	51.8 [44.4–61.5]	54.6 [46.1–62.0]	0.583
*Reason for ECMO therapy* [n (%)]			0.685	
Pulmonary	64 (47.8)	24 (42.1)	88 (46.1)	
Cardiac	56 (41.8)	28 (49.1)	84 (44.0)	
Combined	11 (8.2)	3 (5.3)	14 (7.3)	
Other	3 (2.2)	2 (3.5)	5 (2.6)	
*ECMO type* [n (%)]			0.573	
Veno-arterial	77 (57.5)	31 (54.4)	108 (56.5)	
Veno-venous	55 (41.0)	26 (45.6)	81 (42.4)	
Veno-venoarterial	2 (1.5)	–	2 (1.1)	
*Additional devices* [n (%)]				
Intra-aortic balloon pump	10 (7.5)	6 (10.5)	16 (8.4)	
Impella	5 (3.7)	5 (8.8)	10 (5.2)	
Haemofilter	1 (0.75)	–	1 (0.5)	
*Medical treatment* [n (%)]				
Intubated	124 (92.5)	55 (96.5)	179 (93.7)	
Not intubated	10 (7.5)	2 (3.5)	12 (6.3)	
Vasopressors	111 (82.8)	50 (87.7)	161 (84.3)	
No vasopressors	21 (15.7)	6 (10.5)	27 (14.1)	
Vasopressor missing	2 (1.5)	1 (1.8)	3 (1.6)	
Sedated	119 (88.8)	53 (93)	172 (90.0)	
Not sedated	12 (9.0)	3 (5.3)	15 (7.9)	
Sedation missing	3 (2.2)	1 (1.7)	4 (2.1)	

**Fig. 2:**
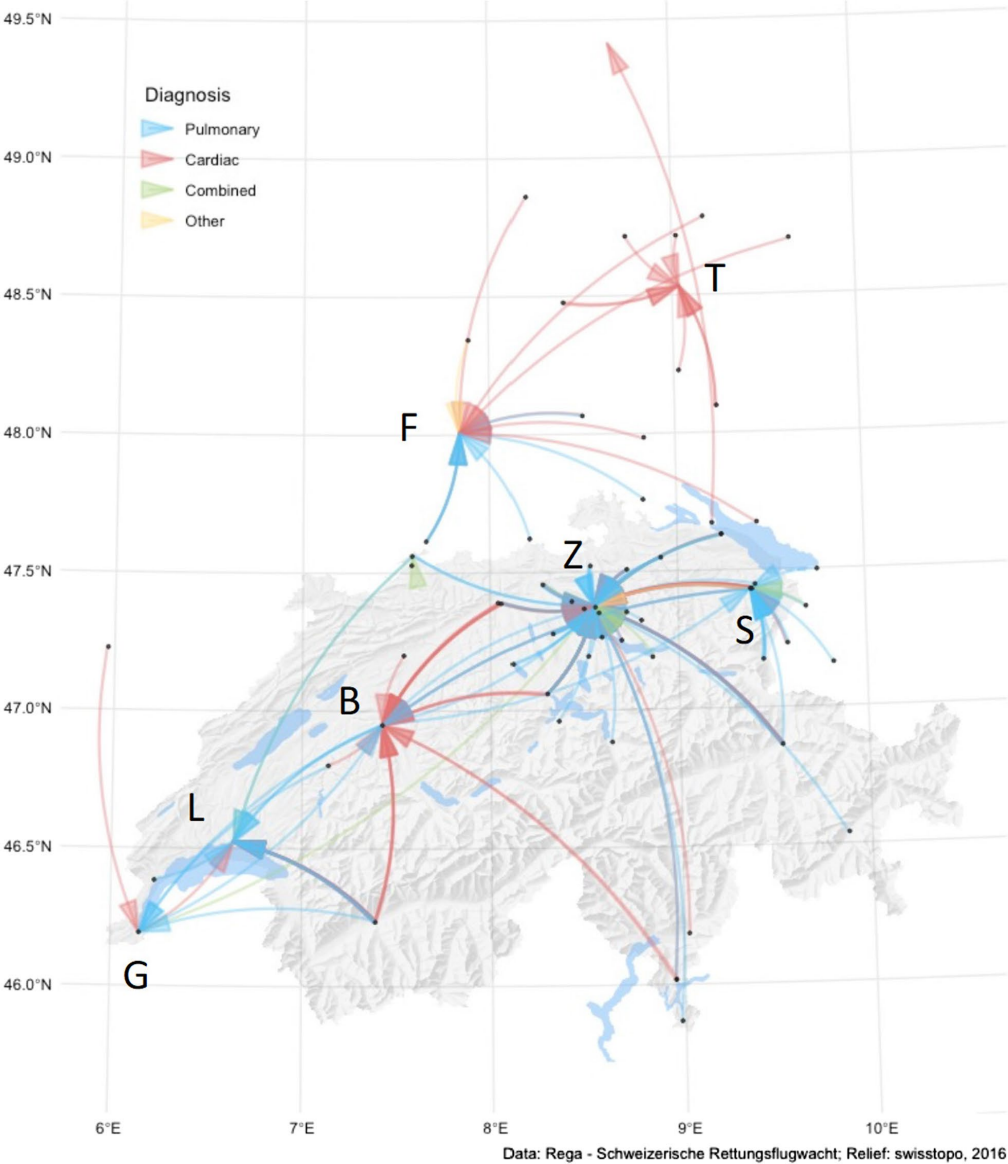
All of the missions carried out by helicopter with a patient under extracorporeal membrane oxygenation. Arrows, colour-coded according the main diagnosis, starting on the map [18] with black dot for referral hospital, pointing towards destination tertiary hospital. Main destination hospitals: B, Bern (Switzerland); F, Freiburg (Germany); G, Geneva (Switzerland); L, Lausanne (Switzerland); S, St. Gallen (Switzerland); T, Tuebingen (Germany); Z, Zurich (Switzerland)

The flight mission details are given in Table 2. Most of the flights were in Switzerland and during the daytime (122/169, 72.2%). Figure 2 shows the geographic details of the missions.

**Table 2:** Mission details for air medical inter-hospital transferred patients under extracorporeal membrane oxygenation

Mission details	Daytime	Night-time	Total	*p*
Numbers [n (%)]	134 (69.8)	57 (30.2)	191 (100.0)	
*Mission classification* [n (%)]				0.109
Primary mission	65 (50.8)	35 (63.6)	100 (54.6)	
Secondary mission	63 (49.2)	20 (36.4)	83 (45.4)	
Missing	6	2	8	
*Mission times* (min; median [Q1–Q3])				
Response time (n = 198)	88.5 [52.7–137.0]	83.0 [49.0–112.0]	85.0 [51.0–135.0]	0.389
Flight time	18.5 [11.2–28.0]	15 [11.0–22.0]	17 [11.0–27.0]	0.209
Handover time (n = 190)	114.0 [72.3–152.8]	112.5 [71.3–176.5]	113.0 [72.0–164.5]	0.534
Total mission time	183.0 [147.0–248.7]	182.0 [131.0–256.0]	182.0 [143.0–253.5]	0.958
Distance (km [range])	58.7 [33.7–75.5]	45.7 [28.9–67.8]	52.7 [33.2–71.1]	0.265
*Referral hospital* [n (%)]				0.374
Swiss hospital	115 (85.8)	46 (80.7)	161 (84.3)	
International hospital	19 (14.2)	11 (19.3)	30 (15.7)	
*Destination hospital* [n (%)]				0.176
Swiss hospitals	122 (91.1)	47 (82.5)	169 (88.5)	
Zurich	74 (55.2)	34 (59.7)	108 (56.6)	
Bern	15 (11.2)	7 (12.3)	22 (11.5)	
Lausanne	19 (14.2)	2 (3.5)	21 (11.0)	
St. Gallen	11 (8.2)	2 (3.5)	13 (6.8)	
Geneva	2 (1.5)	2 (3.5)	4 (2.1)	
Basel	1 (0.8)	–	1 (0.5)	
German hospitals	12 (9.0)	10 (17.5)	22 (11.5)	
Freiburg	6 (4.5)	8 (14.0)	14 (7.3)	
Tuebingen	5 (3.7)	2 (3.5)	7 (3.7)	
Heidelberg	1 (0.7)	–	1 (0.5)	

Response time, from alert of helicopter crew to landing at referral hospital

Flight time, helicopter start at referral hospital with patient to landing at destination hospital

Handover time, helicopter landing at referral hospital to take-off with patient to destination hospital

Total mission time, alert of helicopter crew to landing at destination hospital with patient

### Adverse events

**Fig. 3:**
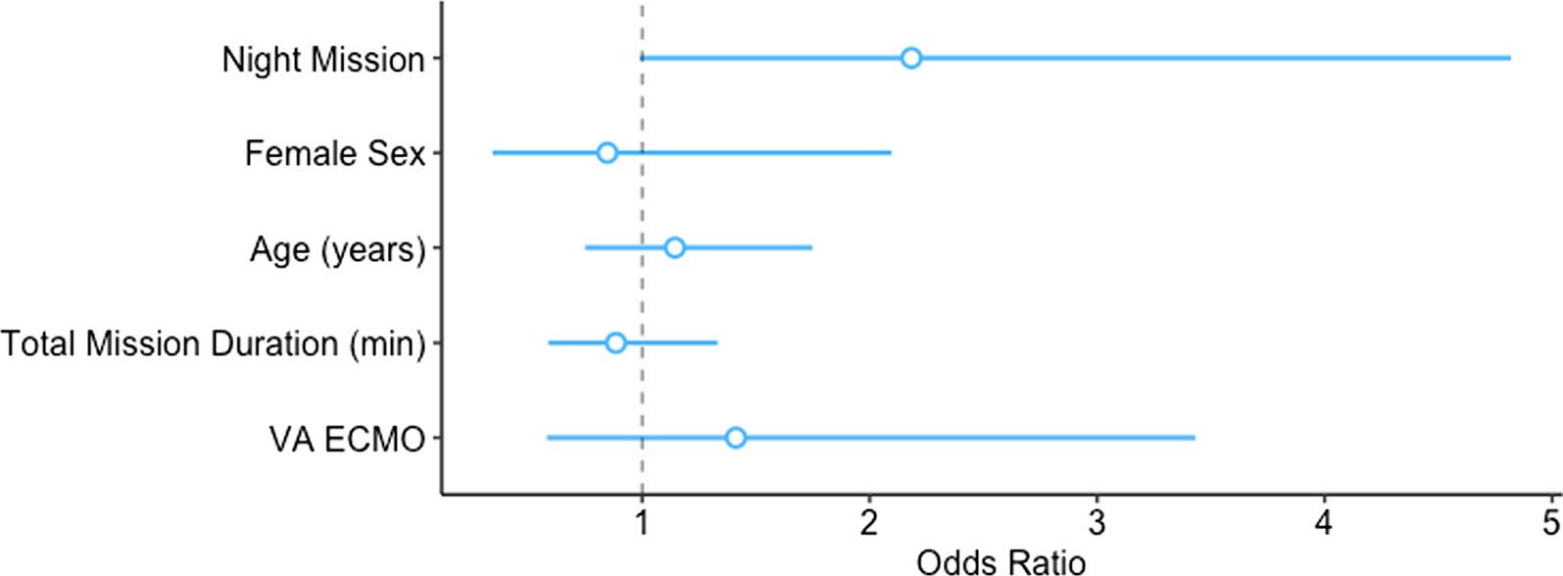
Odds ratios for adverse events shown with regression coefficients (blue circle) and corresponding 95% confidence limits (lines). The variables daytime, veno-venous extracorporeal membrane oxygenation (ECMO) and male sex are reference groups

There were 37 (19.4%) missions with adverse events, of which 7 (18.9%) had more than one event, as given in Table 3. Of these adverse events, two-thirds were considered medical (26, 70.3%), and one-third non-medical (11, 29.7%). Nine (29.1%) of the adverse events were considered to be ECMO related. Two-thirds (6, 66.6%) of these were observed during insertion (e.g., difficult cannulation due to hypovolaemia, severe bleeding with the need for vascular suture), and only one-third (3, 33.3%) during transport (“suck-down” of the ECMO, solved by either reduction of ECMO flow or administration of additional fluids). The medical events were more frequent during the take-over of the patient (e.g., change of syringe pumps). Non-medical events arose from weather-related delays (e.g., landing or take off not possible) and organisational issues (e.g., missing equipment, missing support to unload the patient from the helicopter). We did not observe any adverse events in the sub-cohort of patients under 18 years of age. Adverse events occurred more frequently during night-time missions (16, 28.1%, vs. 21, 15.7% during daytime; *p* = 0.047), as given in Table 3. This was not further confirmed in the logistic regression model, as shown in Fig. 3 and given in Table 4.

**Table 3:** Adverse events and survival for patients undergoing extracorporeal membrane oxygenation (ECMO) transferred by helicopter

Adverse events/survival	Daytime	Night-time	Total	*p*
Numbers	134 (70.2)	57 (29.8)	191 (100)	
*Missions with adverse events*	21 (15.7)	16 (28.1)	37 (19.4)	
Primary missions	9 (45.0)	7 (46.7)	16 (45.7)	
Secondary missions	11 (55.0)	8 (53.3)	19 (54.3)	
Classification missing	1	1	2	
Total adverse events*	25 (100)	19 (100)	44 (100)	
*Medical adverse events*	18 (72.0)	13 (68.4)	31 (70.5)	
Hypotension	6 (33.3)	7 (53.8)	13 (41.9)	
Hypoxemia	3 (16.6)	2 (15.4)	5 (16.1)	
Ventilation	4 (22.2)	–	4 (12.9)	
ECMO related^a^	5 (27.8)	4 (30.8)	9 (29.1)	
During insertion	3 (16.7)	3 (23.1)	6 (19.4)	
During transport	2 (11.1)	1 (7.7)	3 (9.7)	
*Non-medical adverse events*	7 (28.0)	6 (31.6)	13 (29.5)	
Organisation^b^	5 (71.4)	6 (100)	11 (84.6)	
Weather-related^c^ (wind)	2 (28.6)	–	2 (15.4)	
*Patient survival*				
Transport survivors	134 (100)	57 (100)	191 (100)	
*Alive at day 28/ICU discharge*				0.901
Yes	61 (54.5)	25 (55.6)	86 (54.8)	
No	51 (45.6)	20 (44.4)	71 (45.2)	
Missing	22	12	34	
*Alive at hospital discharge*				0.592
Yes	44 (44.9)	19 (50.0)	63 (46.3)	
No	54 (55.1)	19 (50.0)	73 (53.7)	
Missing	36	19	55	

Data presented as n (%)

*One mission can have several adverse events

^a^e.g., difficult cannulation, severe bleeding

^b^e.g., time delay due to ECMO installation in helicopter, installation of intra-aortal balloon pump and ECMO, cannulation needed before transportation, or collaboration with other rescue organisations

^c^e.g., heavy winds, start sometimes impossible because of weather conditions

**Table 4:** Logistic regression model for the odds of an adverse event according to predefined clinical variables

Variable	Odds ratio	95% confidence interval	*p*
*Time*			
Daytime	–	–	
Night-time	2.18	0.98–4.83	0.056
*Sex*			
Male	–	–	
Female	0.85	0.33–2.05	0.719
Age	1.01	0.98–1.04	0.528
Total mission time	1.00	1.00–1.00	0.538
ECMO type*			
Veno-venous	–	–	
Veno-arterial	1.41	0.59–3.50	0.443
*Mission classification*			
Primary mission	–	–	
Secondary mission	1.25	0.55–2.87	0.589

*ECMO type variable grouped as veno-venous and veno-arterial, with veno-venoarterial was merged with veno-arterial due to low numbers (n = 2)

### Survival and follow-up

**Fig. 4:**
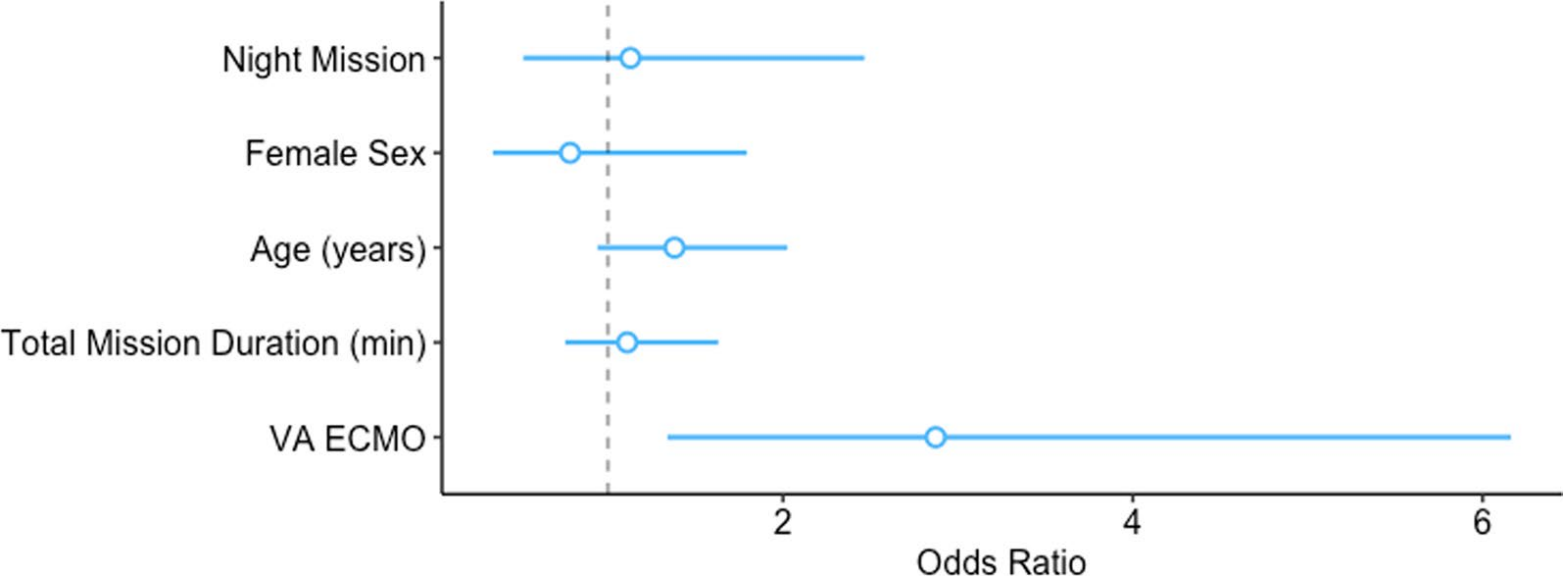
Odds ratios for 28-day survival shown with regression coefficients (blue circle) and corresponding 95% confidence limits (lines). The variables daytime, veno-venous extracorporeal membrane oxygenation (ECMO) and male sex are reference groups

Table 3 gives the survival and follow-up data. All 191 patients survived the transport. Follow-up data for survival to 28 days after the transport or to Intensive Care Unit discharge were available for 157 patients (82.2% of total). The majority of these patients (86, 54.8%) were still alive, including 14 (14/32, 43.8%) of the patients with ECPR. Follow-up data for survival to hospital discharge were available for 136 patients (71.2% of total). Sixty-three of these patients (46.3%) were still alive, including 11 (11/28, 39.3%) of the patients with ECPR. The logistic regression model indicated a higher odds ratio for 28-day survival for patients with a veno-arterial ECMO (2.87; 95% confidence interval, 1.35–6.25; *p* = 0.006), as given in Table 5 and shown in Fig. 4.

**Table 5:** Logistic regression model for the odds ratio of survival at day 28 or to discharge for the 135 available patients after helicopter transfer on extracorporeal membrane oxygenation (ECMO)

Variable	Odds ratio	95% confidence interval	*p*
*Time*			0.762
Daytime	–	–	
Night-time	1.13	0.51–2.48	
*Sex*			0.564
Male	–	–	
Female	0.78	0.34–1.80	
Age	1.02	1.00–1.05	0.092
Total mission time	1.00	1.00–1.00	0.585
ECMO type*			**0.006**
Veno-venous	–	–	
Veno-arterial	2.87	1.35–6.25	
*Mission classification*			0.243
Primary mission	–	–	
Secondary mission	1.56	0.74–3.30	

*ECMO type variable grouped as veno-venous and veno-arterial, with veno-venoarterial was merged with veno-arterial due to low numbers (n = 2), Bold values are statistically significant (p <0.05)

## Discussion

This retrospective analysis of the Swiss Air-Rescue database shows that helicopter transfer of patients undergoing ECMO is safe and that all patients survived the transport, which confirms earlier reports [10, 11]. Medical adverse events were mainly hypotension, and these occurred more often during the night-time missions (28%), while ECMO-related events (e.g., bleeding, difficult cannulation) happened rarely. Non-medical adverse events occurred for one-third of the missions. The 24-h/7-days per week service showed similar handover and mission times between daytime and night-time missions.

### Adverse events

Audits and analysis of adverse events are crucial to improve the operation of clinics and case management and flight logistics, with the aim to guarantee patient safety during these highly specialised services [7]. Our findings of rare adverse events during inter-hospital transfers for these ECMO patients are in-line with previously published incidence reports of adverse events, and they underline the overall safety of the system [10, 11]. All observed ECMO related adverse events are among the known complications for ECMO [1, 2], regardless of the need for a medical transfer. However, HEMS crews need to be aware that these are potential risks also during transfer, to be best prepared and ensure an improved safety culture. We also confirmed that where one adverse event happens, there can also be a second one [10]. Adverse events tended to happen more often during the night-time inter-hospital transfers. This might be explained as reduced cognitive performances [19] and environmental circumstances during the night-time (e.g., darkness), with increased difficulty for efficient trouble management. These circumstances might be trained for in high-fidelity training centres, as already reported for helicopter missions with human external cargo [20]. The logistic regression model indicated night-time missions are not to be associated with more adverse events. Hence, from a clinical point of view, it might be that during night-time, the relatively more critical patients might be transferred, and that the patient condition might lead to an accumulation of adverse events [21]. Organisation of such transport is highly complex, especially for primary missions where fast intervention times are of importance. Some organisational adverse events were identified as communication errors (e.g., patient weight not requested, additional devices not announced). Other challenges of the system here were the compatibility of different ECMO types and additional devices. Indeed, the transport of a patient with ECMO and simultaneous treatment with an intra-aortal balloon pump can only be provided by one specific helicopter base.

### Feasibility and quality of a 24/7 helicopter transfer system

The feasibility, quality and ability to deliver a 24-h/7-days per week helicopter flight service was confirmed by the similar durations of the mission responses, handover times and total mission times, as well as by the similar flight distances between daytime and night-time missions. Taking the specific geographic circumstances of Switzerland into account, with its high Alpine mountains, deep valleys and large numbers of lakes, ground ambulance transfer needs substantially more time even for short distances [22], and therefore the use of a helicopter is reasonable, as recommended by the ELSO [14].

In contrast to other transfer systems, the transfer service here that is operated from different bases is embedded in the well-functioning pre-hospital emergency medical system. This system minimises helicopter maintenance expenses and costs for special trained crews, who are solely deployed for ECMO inter-hospital transfers. The helicopters are staffed with advanced HEMS physicians. These are trained to ensure a continuous evaluation and appropriate treatment of the patient during the mission. Considerations regarding the operational environment (e.g., high altitudes) and the potential influence on medical equipment (e.g., need for recalibration) are part of the training.

However, we can also report here some limiting factors for these inter-hospital transfers through the helicopter system. One patient with a bodyweight of 180 kg could not be loaded into the helicopter due to exceeding the permissible total weight of the load. In this case, the usual inquiry in advance of the patient body weight failed, and he was finally transported by ground ambulance services. Therefore, this case was excluded from the analysis. One primary mission had some time delay because of bad weather conditions during the arrival landing. Therefore, the helicopter could not land on top of the hospital, and instead landed close by, with the team then carried by ambulance on the ground to the final destination. Nevertheless, the final air-medical transport of the patient was without further problems. The weather is a possible system-related factor for ECMO transfer by helicopter, even though this was of low incidence in our analysis and might be generalised to all HEMS missions. These limitations justify the provision of a ground ambulance transfer system as a backup whenever a helicopter transfer is not possible due to such weather-related or organisational issues.

### Survival and follow-up

Patient survival under ECMO treatment in the literature has been reported in a relatively heterogeneous manner, which might be explained by various factors, such as age, case mix and ECMO type. The 28-days survival for the present cohort was a little higher than what was reported in a systematic review [2]. In this review, most patients had a veno-arterial ECMO, while the ECMO type in the present cohort was more homogeneously distributed. Roch et al. reported a rate of 44% hospital survivors in patients transferred on ECMO with acute respiratory distress syndrome [23]. Of the total 142 patients considered for ECMO by the mobile team, only 85 were finally treated with ECMO, and 91% had a veno-venous ECMO. As might have been expected, the study identified age, sepsis-related organ failure assessment score and a diagnosis of influenza as factors in the evaluation of risk of death. Biscotti et al. reported on 100 transported patients with a relatively high survival to 30 days (71%), where 79% of the patients were treated with veno-venous ECMO [9]. Bryner et al. also reported a relatively high survival rate of 70% for patients with respiratory indications and 50% for patients with cardiac indications who were transferred on ECMO [12].

Interpretation of the survival rate in the present study needs to be careful. First, there is the possibility of a selection bias of the patients; e.g., the sicker patients are transferred. According to the medical records of some of the patients, withdrawal of ECMO treatment was decided shortly after transfer to the tertiary centre. These decisions require experience, extended neuro-functional diagnostics, and finally interdisciplinary consensus, which might not be available in all referral hospitals.

### Extracorporeal cardiopulmonary resuscitation

The present cohort included 35 patients with ECPR. Traditionally, survival rates for patients with an in-hospital cardiac arrest treated by conventional cardiopulmonary resuscitation is reported to be around 35% [24], and 10% with an out-of-hospital cardiac arrest [6]. A systematic review of patients with out-of-hospital cardiac treated with ECPR reported survival rates up to 18% [6]. The reported survival rates for patients with ECPR in the present cohort are slightly higher than the survival rate for adults of 41% given in the ELSO registry [4]. However, a systematic review showed that patients who were cannulated with ECMO during refractory cardiac arrest had better neurological survival with protocols using a strict time cut-off from the start of resuscitation to the start of ECMO [25]. In this report, for at least one patient in normothermia, ECMO was only started 2 h after the collapse (1 h pre-hospital, and an additional hour of in-hospital conventional cardiopulmonary resuscitation).

An obvious limitation of the present study is the retrospective character of this analysis, with a possible under-reporting bias of adverse events. Due to the low overall number of adverse events, there is the possibility of random error in the interpretation of the data. Poor data entry occurred due to the heterogeneous medical data documentation of ECMO case details in the transport form, which was performed on the standard helicopter transport form that does not have specific ECMO-related items, like flow, pressure changes, and oxygenator settings and placing of cannulas. Our presented analysis of survival rates might have a rather descriptive character. Findings should be carefully interpreted and might not explain causality. However, as a result of this study, the Swiss Air-Rescue have developed a specific ECMO medical data recording form. Follow-up data on survival were reported with the existing medical reports in the database, which were missing for many cases. For many patients with ECPR, it remained unclear if they had had an in-hospital or out-of-hospital cardiac arrest.

## Conclusions

All patients under ECMO survived the helicopter transport. Adverse events were observed for about 20% of the flight missions, with a tendency during the night-time flights, although none of these harmed the patients. Inter-hospital transfer for patients undergoing ECMO provided by 24-h/7-d per week helicopter emergency medical service teams can be considered as feasible and safe. The majority of the patients (54.8%) were still alive after 28 days.

## Supplementary Information

**Additional file 1**. Main diagnosis according to the International Classification for Disease (ICD-10) for the helicopter transferred patients with ECMO.

## Data Availability

The dataset analysed during the current study is available from the corresponding author upon reasonable request and with permission of the responsible Ethics Committee.

## Abbreviations

ECMO: Extracorporeal membrane oxygenation
ECPR: Extracorporeal cardiopulmonary resuscitation
ELSO: Extracorporeal Life Support Organization
HEMS: Helicopter Emergency Medical Service
