# Supplementary material for: Helicopter inter-hospital transfer for patients undergoing extracorporeal membrane oxygenation: a retrospective 12-year analysis of a service system
Source: Scand J Trauma Resusc Emerg Med. 2022 May 7;30:33. doi: 10.1186/s13049-022-01018-0 (PMC9077885; doi:10.1186/s13049-022-01018-0)
Supplement: Supplementary file 1 — Additional file 1. Main diagnosis according to the International Classification for Disease (ICD-10) for the helicopter transferred patients with ECMO. [file 13049_2022_1018_MOESM1_ESM.docx]

**Supplemental Content 1**

**Table 1.** Main diagnosis according to the International Classification for Disease ICD-10 [16] for the helicopter transferred patients with ECMO

| **Main diagnosis** | **Daytime** | **Night-time** | **Total** | **p** |
| --- | --- | --- | --- | --- |
| Overall | 134 (70.2) | 57 (29.8) | 191 (100) | 0.608 |
| A41.9 | 1 (0.7) | 0 (0.0) | 1 (0.5) |  |
| B37.7 | 0 (0.0) | 1 (1.8) | 1 (0.5) |  |
| I21 | 5 (3.7) | 0 (0.0) | 5 (2.6) |  |
| I21.0 | 2 (1.5) | 0 (0.0) | 2 (1.0) |  |
| I21.1 | 1 (0.7) | 2 (3.5) | 3 (1.6) |  |
| I21.4 | 0 (0.0) | 1 (1.8) | 1 (0.5) |  |
| I21.9 | 17 (12.7) | 5 (8.8) | 22 (11.5) |  |
| I25.14 | 1 (0.7) | 0 (0.0) | 1 (0.5) |  |
| I25.19 | 1 (0.7) | 0 (0.0) | 1 (0.5) |  |
| I26.9 | 2 (1.5) | 1 (1.8) | 3 (1.6) |  |
| I46.0 | 11 (8.2) | 6 (10.5) | 17 (8.9) |  |
| I50.01 | 5 (3.7) | 3 (5.3) | 8 (4.2) |  |
| I50.14 | 1 (0.7) | 0 (0.0) | 1 (0.5) |  |
| I71.00 | 1 (0.7) | 0 (0.0) | 1 (0.5) |  |
| J10.0 | 1 (0.7) | 0 (0.0) | 1 (0.5) |  |
| J10.1 | 1 (0.7) | 0 (0.0) | 1 (0.5) |  |
| J18.9 | 1 (0.7) | 0 (0.0) | 1 (0.5) |  |
| J18.98 | 1 (0.7) | 0 (0.0) | 1 (0.5) |  |
| J44.90 | 1 (0.7) | 0 (0.0) | 1 (0.5) |  |
| J80 | 38 (28.4) | 18 (31.6) | 56 (29.3) |  |
| J80.U07.1 | 14 (10.4) | 4 (7.0) | 18 (9.4) |  |
| J84.1 | 1 (0.7) | 0 (0.0) | 1 (0.5) |  |
| J96.00 | 2 (1.5) | 0 (0.0) | 2 (1.0) |  |
| R57.0 | 16 (11.9) | 12 (21.1) | 28 (14.7) |  |
| R57.1 | 0 (0.0) | 1 (1.8) | 1 (0.5) |  |
| R57.2 | 7 (5.2) | 1 (1.8) | 8 (4.2) |  |
| T07 | 1 (0.7) | 0 (0.0) | 1 (0.5) |  |
| T62.2 | 0 (0.0) | 1 (1.8) | 1 (0.5) |  |
| T75.1 | 2 (1.5) | 1 (1.8) | 3 (1.6) |  |

Data presented as n (%)
